# Bridging the Gap in Periodontal Health Knowledge: A Comprehensive Review of Awareness Levels and Barriers in Saudi Arabia

**DOI:** 10.7759/cureus.90662

**Published:** 2025-08-21

**Authors:** Khalid Alkhurayji, Yousef Shabi, Abdulrahman Aloqayli, Mohammed Alsadoon, Ahmed Alabdullah, Muteb Almuways, Saud Alharbi, Naif Alqahtani

**Affiliations:** 1 College of Public Health, Health Information Management and Technology, Imam Abdulrahman Bin Faisal University, Riyadh, SAU; 2 Dentistry, Prince Sultan Military Medical City, Riyadh, SAU; 3 Dentistry, King Abdulaziz Naval Base Armed Forces Hospital, Jubail, SAU

**Keywords:** clinical dentistry, dental practice, health education & awareness, periodontal disease, public health

## Abstract

Understanding of periodontal diseases and their broader health relevance is considered an important aspect to achieve better self-awareness of health. Therefore, this review aimed to explore the awareness levels of periodontium diseases among various locations and populations in Saudi Arabia. A comprehensive approach was used among multiple databases and search engines, including PubMed, Web of Science, Scopus, and Google Scholar, focusing on awareness levels among the general population and vulnerable groups (such as diabetic patients and pregnant women), in addition to healthcare providers. The synthesis of the review follows the hermeneutic approach, which involves identifying themes through repeated reading and interpretation of the findings. The review shows the urgent need for culturally sensitive and comprehensive oral health educational programs targeting the general population, vulnerable groups, and healthcare providers in Saudi Arabia. Integration of educational oral health programs into the routine practice of maternal health management and chronic disease clinics, along with prevention campaigns and healthcare provider training and development, is essential to improve the level of awareness of periodontal diseases.

## Introduction and background

Periodontal diseases are widely recognized, impacting the surrounding tissue of the tooth, including the periodontal ligament, supporting structures of the teeth, gums, and alveolar bone, with potential harm that could go beyond periodontal health [1]. Recently, scientific studies have demonstrated the link between periodontal diseases and several systemic illnesses such as diabetes mellitus, metabolic syndrome, adverse pregnancy outcomes, respiratory diseases, and cardiovascular disease [2]. These associations can act as risk factors for the progression of systemic illnesses, which makes oral health a vital component of overall health [3].

In the context of Saudi Arabia, chronic disease prevalence, such as diabetes and cardiovascular conditions, was reported to be high compared to other countries, which has led to increased attention on prevention measures under the 2030 vision transformation plan of the health sector [4,5]. Unfortunately, the awareness level of periodontal diseases, particularly in terms of systemic consequences, remained limited and low among the general population and the specific vulnerable population. Poor oral hygiene practice, limited access, and insufficient integration in terms of educational prevention programs in healthcare services could contribute to hindering the improvement in these areas [6].

Previous studies indicated that awareness of periodontal diseases and their relationship to systemic health is low in both the general population and within vulnerable patients. These investigations emphasize the need for ongoing future research to bridge the knowledge gap and promote preventative strategies through comprehensive approaches [3,6].

A comprehensive and holistic understanding of the population, along with healthcare providers' awareness, is considered an important aspect for the development of effective prevention initiatives and campaigns. Previous investigations have made an effort to assess the awareness level regarding periodontal diseases and the links with systemic illness among different settings, regions, and populations in Saudi Arabia. However, the levels of awareness remain low, with significant variation across populations such as the general public, diabetes patients, pregnant women, dental practitioners, and caregivers of individuals with special needs. Therefore, this review aimed to explore the awareness levels of periodontium diseases among various locations and populations in Saudi Arabia.

## Review

Methods

Research Design

This review was conducted using a comprehensive integrative approach to summarize and interpret the awareness level of periodontal diseases in the context of Saudi Arabia. This study was guided by the patient, intervention, comparison, and outcome (PICO) framework in order to define the scope of the exclusions and inclusion criteria [7].

In terms of population items, the population residing in any region of Saudi Arabia, such as the general public, dental patients, diabetic patients, caregivers, pregnant women, and healthcare providers, was included. While the exposure of intense included the assessment or knowledge of periodontal diseases and/or the link assessed with systemic conditions. However, a comparator was required due to the nature of the necessity of the included studies and the subgroup comparisons, such as education, profession, and/or region, that were recorded when obtainable. The outcome of interest in this study includes the level of awareness experienced in category responses, percentage, and scores, while the secondary outcome includes influencing factors and/or reported barriers.

Search Strategy

The search was applied in four search engines and databases, which include Google Scholar, Web of Science, PubMed, and Scopus. The search strategy focused on identifying research reported and related to periodontal disease awareness in the context of Saudi Arabia. The Boolean operators help to ensure that the terms used provide a comprehensive and accurate search strategy. The keywords used in this study were "periodontal disease," "periodontitis," and "gingivitis" in addition to awareness-related terms including "awareness," "knowledge," "perception," and "attitude"; systemic health conditions such as "systemic disease," "systemic health," "diabetes," "pregnancy," "cardiovascular disease," "heart disease," "metabolic syndrome," and "chronic disease"; and lastly the geographical identifiers like "Saudi Arabia," "KSA," and "Kingdom of Saudi Arabia." Searches were adapted to suit the indexing and capabilities of each database. No language restrictions were applied in the search strategy to ensure comprehensive inclusion of relevant studies. However, studies published before 2015 were excluded in order to focus on the most recent and relevant evidence.

Inclusion and Exclusion Criteria

Any observational studies conducted in the context of Saudi Arabia were eligible for this review, which reported and measured the knowledge or awareness level regarding periodontal diseases. Furthermore, studies executed outside Saudi Arabia and/or involving populations not relevant to the scope of the study or that did not assess knowledge or awareness of periodontal disease were excluded. However, studies that do not report baseline awareness data, conference abstracts, and/or related information in the context of Saudi Arabia were excluded.

Data Extraction

The extraction of data was conducted using a structured form, which included study characteristics such as region and population, in addition to the awareness level, type, and reported barriers. However, due to variation in the tools used in assessing the awareness levels, a narrative synthesis was used in this study. Any disagreements among authors during screening or data extraction were addressed through discussion and consensus.

Synthesis of the Studies

Following the hermeneutic synthesis approach [8], the theme was reported in this review throughout the reading and interpretation of the study findings, while the quantitative data were summarized descriptively in terms of count and percentage as appropriate. This combined approach allowed for a more nuanced understanding of the evidence while accommodating the methodological heterogeneity of the included studies.

Results 

Table 1 illustrates the studies among several regions in the context of Saudi Arabia, which indicates that the awareness level of periodontal diseases and the links to systemic health issues are reported to be low to moderate across the population groups. The population in Saudi Arabia is not fully aware of the link between periodontal diseases and systemic illnesses such as metabolic syndrome, diabetes, and adverse pregnancy outcomes such as premature birth and low birth weight. For instance, less than 40% of the population recognizes the link between periodontal disease and systemic illnesses. In addition to that, fewer people know how oral health can impact pregnancy or metabolic conditions [9-14].

**Table 1:** Characteristics of the studies included MetS: metabolic syndrome

Reference	Year	Region (KSA)	Population	Study Design	Awareness Focus	Awareness Level	Main Findings	Barriers to Awareness
[9]	2024	Not-specific	General population	Cross-sectional	Link between periodontal and systemic diseases	Only 38.82% of participants recognized the association between periodontal diseases and systemic diseases, while 60.3% reported the need for periodontal checkups for individuals with systemic diseases.	The study highlighted a particularly low awareness of the associations between periodontal diseases and other systemic conditions such as infertility, stroke, and metabolic diseases.	Deficiency in educational initiatives and public oral health campaigns.
[10]	2020	Jizan	General population	Cross-sectional	Awareness of periodontal treatment	Approximately 45.9% of participants believe that periodontal disease is genetic, and only 26.5% were knowledgeable about the link between low birth weight and premature births.	Most participants were unaware of the importance and procedures related to periodontal surgery.	Poor knowledge and attitude toward periodontal care.
[11]	2015	Jeddah	Diabetic patients	Cross-sectional	Diabetes and periodontal disease	A mere 21.8% understood that gum disease can make it harder to control blood sugar levels in diabetic patients.	Diabetic patients underestimated the connection between diabetes and gum health.	Lack of structured patient education and provider guidance.
[12]	2023	Asir	Diabetic patients	Cross-sectional	Periodontal health importance	The awareness level regarding the bidirectional relationship between diabetes and periodontal diseases among diabetic patients in the Asir region was found to be high in 43% of the participants.	Participants acknowledged the connection but couldn't explain the mechanisms.	Limited integration of dental-health topics in chronic disease education.
[13]	2023	Qassim	Dental patients	Cross-sectional	Relationship between periodontal and systemic health	The overall awareness level among all study subjects regarding the relationship between periodontal disease and systemic health was found to be 52.3%, which is considered average	Awareness is highest for anemia (63%), lower for diabetes (48%), and respiratory conditions (17%).	Lack of knowledge on the diabetes-periodontal link affects compliance and preventive care.
[14]	2024	Riyadh	Pregnant women	Cross-sectional	Impact of periodontal disease on pregnancy outcomes	Only 29.5% of participants were knowledgeable about the link between low birth weight and premature births and periodontal disease.	Awareness is significantly linked to education and trimester; women with secondary education are more likely to seek care.	Fear, low perceived need, cost, and educational disparities.
[15]	2024	Riyadh	Dental practitioners	Cross-sectional	Link between metabolic syndrome and periodontal disease	The mean awareness score for the relationship between MetS and periodontal disease among the practitioners was 3.55 ± 1.73.	Practitioners showed partial understanding of systemic associations with periodontal disease.	Lack of continuing professional education.
[16]	2018	Jeddah	Healthcare providers	Cross-sectional	General periodontal health	Groups were aware that diabetes affects periodontal health, with 96.8% of dentists and 95.7% of physicians acknowledging this.	Oral health complications were more common in less-aware participants.	Health education is not reaching diabetic patients sufficiently.
[17]	2021	Taif	General dentists	Cross-sectional	Dentists' knowledge and referral practices	A high percentage (78%) of general dentists were aware of the signs and symptoms of periodontal disease	Dentists in Taif demonstrated high awareness of periodontal disease signs, symptoms, and risk factors.	Gaps in referral practices, especially for phase-1 therapy.
[18]	2019	Asser	Caregivers and special needs patients	Cross-sectional	Risk factors and signs of periodontal disease	Caregivers in group II (moderate mental retardation) demonstrated the highest awareness, with 74.7% answering correctly about oral health and periodontal knowledge.	Most could not identify the early signs of gum disease.	Not specified clearly in the study.
[19]	2015	Eastern Province	Dental patients	Cross-sectional	Periodontal disease	Only 21% of the participants could correctly define dental plaque. Approximately 30% were aware of the harmful effects of dental plaque.	Participants often did not link bad breath to gum health.	Cultural sensitivity and lack of public health communication.
[20]	2020	Not-specific	General population	Cross-sectional	Periodontal treatment and prevention	More than half of the sample (51.3%) apply basic oral health concepts, such as twice daily tooth brushing and a balanced diet.	Dental patients often confused gum bleeding with brushing technique issues.	Misconceptions about gum symptoms and prevention.
[21]	2020	Riyadh	General population	Cross-sectional	Awareness campaign impact	There was a low level of awareness concerning the exact etiology of periodontal disease, with about 65% of respondents unaware that plaque causes the disease.	Post-campaign, awareness and attitudes toward gum health improved.	Need for more frequent and consistent campaigns.
[22]	2017	Asser	General population	Cross-sectional	Knowledge of periodontal signs	A majority of participants (91.4%) believed that periodontal disease does not require treatment, despite 70% acknowledging that it could lead to tooth loss.	91.4% acknowledged the importance of gum disease but lacked action.	Misunderstanding of bleeding gums as non-serious.

Awareness Among Healthcare Providers

Dental practitioners and healthcare providers revealed a higher level of knowledge in terms of periodontal diseases and the systemic implications, while the general population showed a low level of knowledge. Nonetheless, significant knowledge gaps still persist among healthcare providers, such as the appropriate protocols for referral and treatment methods. For instance, some healthcare providers demonstrated a partial understanding of how metabolic syndrome is connected to periodontal diseases, highlighting the need for the development of professional training and education [15-17].

Knowledge in Vulnerable Groups

Vulnerable populations, such as pregnant women, diabetic patients, and caregivers of special needs patients, usually exhibit a low level of awareness regarding periodontal diseases and the systemic links. This lack of knowledge is influenced by certain factors such as education level, cultural beliefs, and access to reliable health information. To illustrate the case, the diabetic population revealed a lack of understanding regarding the relationship between periodontal disease and diabetes, while pregnant women showed low awareness in regard to periodontal disease and its effect on pregnancy outcomes. Special needs patients, on the other hand, revealed that patients with moderate mental retardation showed relatively better knowledge but also still faced challenges in terms of identifying early signs of periodontal diseases [11,12,14,18].

Barriers to Awareness and Care

Several barriers are reported to the low level of awareness and suboptimal health behaviors that are observed in Saudi Arabia, which include insufficient public education and campaigns dedicated to oral health. Furthermore, a lack of continuing training programs for healthcare providers and integration of oral health for chronic disease management clinics [9,10,15,19-22], in addition to a widely spread concerning issue, such as misunderstanding of periodontal disease symptoms, which include gum bleeding, and the non-serious issue, along with cultural sensitivity that collectively impede the proper seeking of oral health behavior. The reported socioeconomic barriers, such as cost, educational disparities, and fear of dental treatment, further limited patient engagement, particularly among diabetic and pregnant women, which limits the prevention and management effort [14].

Impact of Awareness Campaigns

Targeted awareness campaigns demonstrated the huge ability towards improvement of knowledge and awareness of periodontal diseases. For instance, the post-campaign assessment in Riyadh showed increased awareness regarding the causes of periodontal diseases and the importance of oral health [21]. Despite the positive impact, these campaigns required regular repetition, integration with healthcare services, and regional tailoring in order to expand their impact effectively.

**Figure 1:**
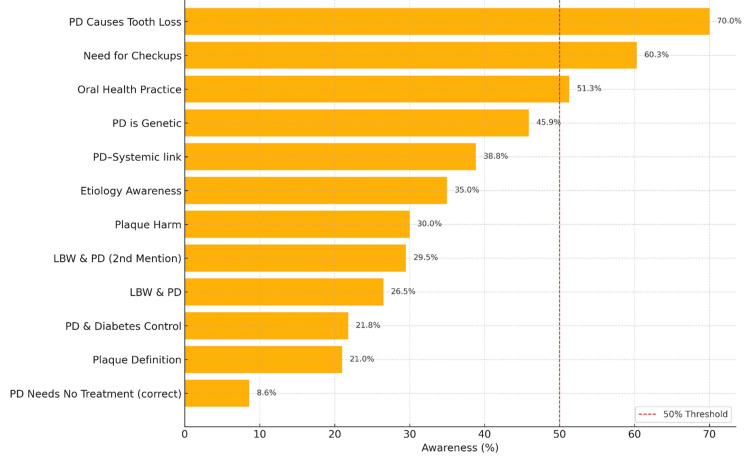
Awareness levels regarding periodontal disease (PD) Created by the authors of this study. LBW: low birth weight

Figure 1 shows the level of population awareness regarding periodontal diseases, which revealed significant awareness gaps. For instance, a high level of awareness is preserved in the case of tooth loss (70.0%), the recognition of the need for dental checkups (60.3%), and the practices of oral health (51.3%). Despite the high levels in these aspects, the awareness level drops notably below 50% in terms of more specific systemic conditions. For instance, fewer people understand that periodontal diseases can be genetic (45.9%) or linked to systemic illnesses (38.8%), while 21.8% recognize the link between diabetes and periodontal diseases. Alarmingly, only 8.6% correctly identified that periodontal disease requires treatment. Comparably, awareness of the link between low birth weight and periodontal diseases ranged between 26.5% and 29.5%, while the harms and definitions of plaque were reported to be between 21.0% and 30.0%.

**Figure 2:**
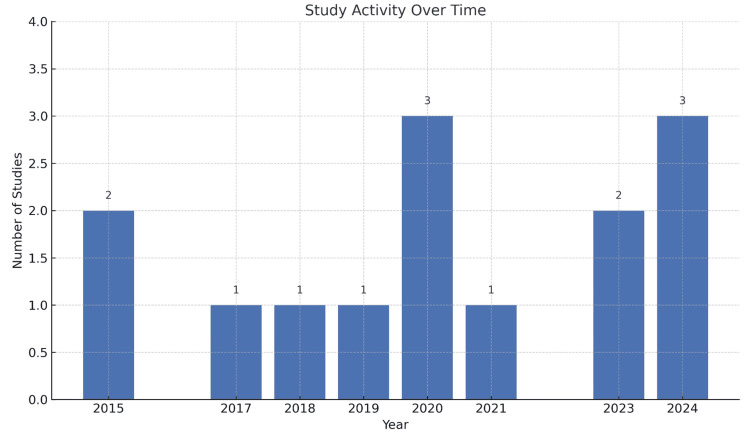
Study activity regarding periodontal awareness over time Created by the authors of this study.

Figure 2 shows the studies conducted over time from the baseline of 2015. The research activity appears to be sporadic, with notable peaks in 2020 and 2024. While moderate activity was observed in 2015 and 2023, with two studies each year. However, in the years 2017, 20018, 2019, and 2021 reported by only one study reported per year.

**Figure 3:**
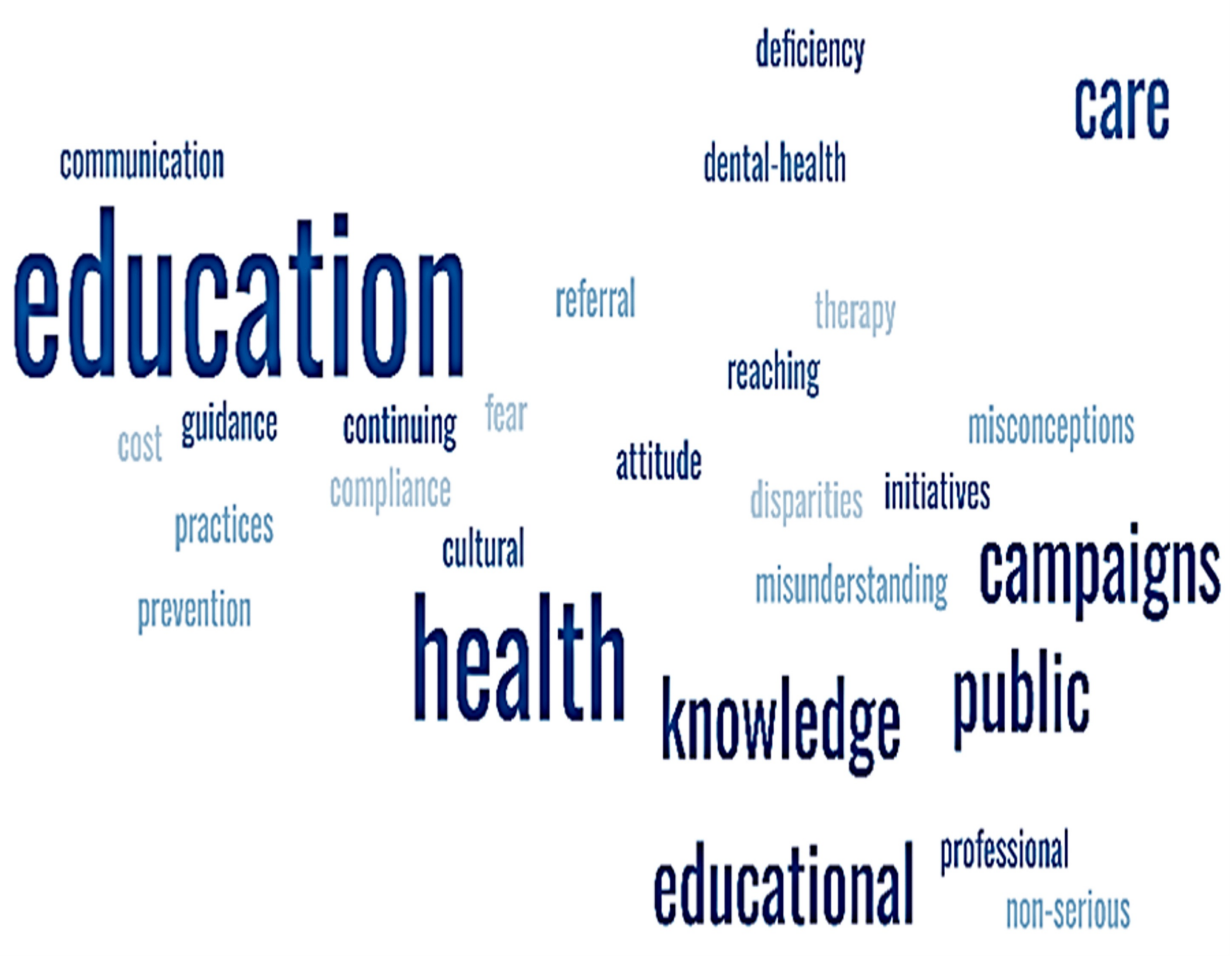
Word cloud related to public knowledge and awareness Created by the authors of this study through www.freewordcloudgenerator.com.

Figure 3 illustrates the word cloud, which highlights the key words related to public knowledge and awareness in terms of preventive health, with education emerging as the most repeated word, underscoring the role in improving prevention and awareness. Words such as "misconceptions," "cultural," "compliance," and "referral" pointed to barriers that are in the process of improving knowledge and awareness. This visualization suggested that these terms are essential for awareness and identifying the barriers to increasing knowledge.

Discussion

This study identifies the critical gaps in knowledge and awareness of periodontal diseases and systemic conditions among diverse groups of the population in Saudi Arabia. Despite the fact that periodontal diseases are a prevalent and established link to systemic illnesses such as adverse pregnancy outcomes, diabetes, and metabolic syndrome, awareness levels remained suboptimal, in particular among the general public and vulnerable people such as pregnant women and diabetic patients. These findings align with international studies demonstrating that public awareness of periodontal diseases is frequently limited, which adversely impacts early diagnoses, management, and prevention [1,23].

Regions in Saudi Arabia consistently demonstrate moderate to low levels of awareness in terms of periodontal diseases. The findings reveal a persistent lack of knowledge about periodontal treatment, prevention, and signs of disease, despite the differences in regions of Saudi Arabia, among the general population, such as Jizan, Asser, and other non-specific regions [10,18]. This suggests that despite the different geographical settings, population awareness remained inadequate, which underlines the need for national initiatives to implement widespread and standardized campaigns related to periodontal health.

Comparing urban and rural disparities in terms of periodontal disease awareness showed that populations in urban regions usually have better prevention programs, access to dental care, and health education. However, rural regions reported a limited awareness level, which reflects the urgency and need for improvement in terms of service utilization. These disparities undermine the importance of health educational services in addition to outreach methods for rural regions, such as mobile dental units and integration of oral health education into primary care services, in order to bridge the gaps and improve equitable information and care for the populations [10,15].

In terms of gender-based disparities, awareness level could be impacted, particularly among pregnant women, who showed a concerning level of knowledge about the connection between hormonal changes during pregnancy and the progression of periodontal diseases. Initiatives should highlight the differences and risks of untreated periodontal diseases during pregnancy in order to empower women with actionable knowledge and timely interventions. In addition to accessible care, culturally appropriate educational information, and oral health sessions or counseling through the collaboration between dental and maternal clinics in order to improve awareness and mitigate risk [9,14].

Many healthcare providers in Saudi Arabia usually demonstrate a better level of knowledge regarding oral health compared to the general population. However, gaps persist across this group, given that partial understanding of the complex relationship of periodontal diseases and the inconsistent referral practice, suggesting better initiatives through continuing perinatal education and standardized clinical practices. These study results are consistent with prior research, indicating that enhancement in provider knowledge is essential to facilitating real prevention and intervention through multidisciplinary care [24].

Vulnerable populations such as pregnant women and diabetic patients are considered high-risk groups given the low level of awareness and understanding of periodontal disease relationships with diabetes and the impact on the pregnancy outcome, which may contribute to poorer health. Nonetheless, educational disparities, limited access, and cultural beliefs may tailor health information and likely exacerbate these gaps. These results underscore the necessity of culturally sensitive education interventions in addition to integrated oral health counseling in maternal and chronic health programs [14,25].

Certain barriers were identified across the studies, including misconceptions, socioeconomic factors, and insufficient targeted campaigns, which are consistent with previous investigations reported in other studies [6,26]. The barriers identified can hinder self-care and healthcare service seeking, emphasizing the necessity for strategic interventions in public health. For instance, a study conducted in the Riyadh region showed promising results in improving knowledge and awareness, in addition to attitudes, through health campaigns, which suggests that these methods can play a pivotal role in improving awareness [27].

Comparing the level of awareness in Saudi Arabia with other countries showed significant similarity, in particular among nations with well-established health educational programs. For instance, investigations in countries such as the United States and Sweden revealed low levels of awareness regarding periodontal disease among the general population. More than half of the adults surveyed across these countries were not aware of periodontal disease complications. Furthermore, in the United States, national campaigns of oral health and routine dental visits were common [28,29]. Comparably, a study in Japan indicates a better level of awareness regarding the impact of periodontal disease, specifically in relation to systemic illnesses [30]. 

This variation could be attributed to differences in the delivery of oral health mechanisms, such as the integration of health education programs with healthcare systems and routine dental care visits. While in Saudi Arabia, oral health education programs are usually limited and lack widespread reach across regions, particularly in rural areas [31]. Moreover, across many developed countries, school-based dental programs, routine media campaigns, and community outreach programs are implemented, which can help reduce misconceptions and cultural factors that contribute to knowledge gaps. Nonetheless, these approaches also contribute to early prevention and detection of periodontal disease [32]. 

Healthcare providers in countries such as the United Kingdom and Australia reported routine examination and screening of periodontal health during general health checkups and emphasized dental education during consultations [33]. However, in the context of Saudi Arabia, studies across dental care providers showed a moderate knowledge level, with no consistent counseling regarding periodontal conditions [16]. 

The implication of periodontal disease on the overall health in the context of Saudi Arabia, where the prevalence rate is very high, not only poses a serious challenge in public health but also highlights the urgent need for comprehensive educational support aiming to improve awareness among the population [13]. Recent investigations among many patients revealed alarming statistics: nearly half of adults visiting dentists show signs of periodontitis, reflecting the necessity for both proper hygiene and educational practices improvement [34,35]. Healthcare providers empowering patients to take proactive interventions to improve their oral health can ultimately contribute to enhanced management of diseases and improve the quality of life.

In order to combat the rising issue of non-communicable diseases in Saudi Arabia, public awareness alone is not enough, given that healthcare strategies across the healthcare system should be integrated with health education. Healthcare providers can leverage the outreach program towards the population as evidence of the association between periodontal diseases and other systemic illnesses, such as diabetes and cardiovascular diseases, which contain common pathways of inflammation and risk factors [13,36]. Therefore, collaboration between physicians and dentists could facilitate a more holistic approach towards the healthcare service and patient care, ensuring that populations with systemic illnesses receive complete periodontal evaluation and are integrated into routine health screening. This integrated method can serve to promote the culture of preventive care and aid the advancement of interventions for both periodontal diseases and systemic illness. Despite the advancement of solutions, the global burden of periodontal diseases is modified by socioeconomic factors, as highlighted in recent studies. Nonetheless, the prevalence of periodontal disease costs billions annually due to the disability and healthcare expenses, in addition to the loss of productivity [37].

The economic impact revealed the necessity of integrating national-level intervention strategies through early diagnosis and prevention as key elements. As such, public health interventions should empower educational campaigns that inform patients about the consequences of periodontal disease. In particular, with relation to chronic diseases such as cardiovascular disease and diabetes, which are reported to be high in the context of Saudi Arabia, and associated with poor periodontal health [38]. Therefore, addressing these health-related domains and economic situations enables stakeholders to advocate for public health policies that advance comprehensive treatment and prevention of oral care. Additionally, a closer examination of the associated risk factors that are usually linked with lifestyle and environmental determinants is required. For instance, certain behaviors and attitudes, such as poor oral hygiene practices, tobacco smoking, and poor dietary intake, can trigger the development of periodontal disease, which results in socioeconomic disadvantage among the population [39]. These relationships between periodontal diseases and systemic conditions, such as diabetes, not only increase the risk of diabetes complications. In fact, poor control of diabetes can worsen periodontal health further, which necessitates integrated healthcare service approaches [13]. This suggests that public health initiatives also need to educate about lifestyle modification, which fosters comprehensive strategies to combat systemic illness and periodontal diseases.

In light of these complexities, the role of healthcare professionals in diagnosing and managing periodontal disease becomes increasingly critical. Regular screenings and assessments for periodontal health can not only aid in early detection but also serve as an opportunity to educate patients about the interconnections between their oral health and systemic conditions. For instance, studies have shown that individuals with diabetes who receive periodontal therapy can achieve better diabetes control, highlighting the potential for integrated treatment approaches to yield significant health benefits [40]. Furthermore, implementing standardized protocols such as regular screening in primary care settings can enhance the identification of periodontal disease, leading to improvements in population health management and outcomes [41]. As a result, creating collaborative environmental frameworks between healthcare providers in addressing dual challenges between periodontal diseases and systemic illnesses.

Certain dental care techniques and technologies pose a significant and promising future for the enhancement of disease management and education, such as telehealth services, which can facilitate remote and rural areas, allowing healthcare providers to reach out to underserved patients, thereby increasing access to information on periodontal health and improving awareness. Nonetheless, the application of mobile health can be improved through platforms for transportation of educational resources and reminders of oral health practices, which are considered important for the prevention and early detection of periodontal diseases. Despite these technological advancements, many populations remain unaware of periodontal disease conditions, and leveraging these technologies may improve awareness. However, other aspects need to be considered throughout the culture of proactive planning for population health management across regions [9].

The cultural and social factors are considered an essential aspect for more effective public health intervention in oral health in the context of Saudi Arabia, given the common cultural beliefs and practices that may influence healthy behavior. While awareness is an essential aspect of prevention, healthy behaviors play a major role in terms of oral hygiene, daily practice, and routine clinical visits and dental checkups that can reduce the onset of periodontal disease effects. To illustrate the case, the traditional oral hygiene point of view may promote cosmetic concerns over preventative management, which neglects the underlying health issues. In addition, societal disparities often limit access to dental services, particularly among low-income groups, which face greater barriers to care [42].

While government initiatives play a major role, community engagement in improving oral health awareness cannot be denied. Evidence suggests that community-based interventions in terms of health campaigns and workshops can improve health-seeking behaviors in addition to the improvement of knowledge, particularly among populations with low awareness and knowledge levels [42]. While healthcare providers' role goes beyond the management and prevention, through pivotal advocacy of policy changes and recommendations that promote oral health within the healthcare provider framework. Recent studies showed that collaboration between physicians and dentists can elevate the quality and health outcomes of patients towards the standard of care, particularly among vulnerable patients such as those with diabetes and cardiovascular diseases [43].

Integrating oral health into overall health policies can improve different populations and promote health outcomes, and reduce the economic burden of periodontal disease on the healthcare system. Therefore, involved communities, patients, and local authorities can influence and help tailor measures that improve awareness in the context of Saudi Arabia, which leads to greater acceptance and participation in oral health initiatives. By empowering the community through diverse approaches, stakeholders can address the social determinants of health that contribute to periodontal diseases, paving the way for improvements in healthcare outcomes in Saudi Arabia. These practices could mitigate the prevalence of periodontal diseases and risk awareness among populations.

The study limitations of this review include the predominant cross-sectional designs, which may limit the causal inferences of the findings. The diversity of the population and regions across the studies could affect the generalizability of the results. Despite these limitations, this study synthesizes the findings from different regions and populations within Saudi Arabia, affording a wide-ranging national perception. Including both healthcare providers and the general public, which allows for a well-adjusted understanding of the awareness gaps and barriers. The inclusion of recent studies in Saudi Arabia ensures that the results reflect current opportunities and challenges regarding periodontal awareness.

The implication of this study for clinical practice and public health policy can be observed in the cultural sensitivity of oral health education targeting healthcare providers and the general public. Nonetheless, healthcare providers require professional development and training in addition to clear guidelines to improve the prevention and referral practices. Health camps should be tailored regionally and designed to address socioeconomic and cultural barriers in the context of Saudi Arabia. Future studies should evaluate the effectiveness of interventions and examine new methods, such as digital education, to improve the outreach and engagement.

## Conclusions

This review revealed a low level of awareness of periodontal diseases with the links to systemic complications that remained insufficient among vulnerable patients and the general population in Saudi Arabia, and substantial barriers were observed, including limited education, cultural factors, socioeconomic status, and misconceptions, which hinder effective management and prevention of periodontal diseases. Despite these barriers, culturally sensitive educational health programs targeting both the public and healthcare providers are urgently needed. Integration of oral health programs within chronic disease and maternal health clinics could improve health outcomes. In addition, strengthening healthcare provider training and healthcare policy can further support early detection and multidisciplinary care with coordinated efforts to overcome these barriers and reduce the burden of preventable diseases.
